# Case report: Diarrhea-associated hemolytic uremic syndrome in the Era of COVID-19

**DOI:** 10.3389/fped.2022.979850

**Published:** 2022-10-31

**Authors:** Gina M. Richardson, Sharon W. Su, Sandra Iragorri

**Affiliations:** ^1^ Oregon Health & Science University, Portland, OR, United States; ^2^ Department of Pediatric Nephrology, Randall Children's Hospital at Legacy Emanuel, Portland, OR, United States

**Keywords:** COVID-19, Shiga toxin-producing *E. coli*, hemolytic uremic syndrome, acute kideny injury, thrombotic microagiopathy, case report

## Abstract

Over the past two years, a growing number of SARS-CoV-2 infection-associated clinical pediatric phenotypes have been identified, including a hemolytic uremic syndrome (HUS) form of thrombotic microangiopathy. Oregon’s high prevalence of Shiga toxin-producing *Escherichia coli* (STEC) infections gives it a unique perspective to discuss the impact of COVID-19 and HUS. We seek to highlight SARS-CoV-2 as a potential new infectious etiology of severe diarrhea-associated HUS, based on two cases from Portland, Oregon, occurring in non-COVID-19 immunized children. The first case is a previously healthy ten-year-old who presented with SARS-CoV-2 infection and bloody diarrhea after an appendectomy, followed by full-blown oligo-anuric HUS. Second is a previously healthy six-year-old who presented with short-lived bloody diarrhea, rapidly evolving to HUS, and who tested positive for COVID-19 via polymerase chain reaction and STEC toxins one and two. These two cases highlight two main points. First, SARS-CoV-2 must be included in the differential diagnosis of diarrhea-associated HUS, either as the sole agent or concurrent with a STEC infection. Second, when managing STEC gastroenteritis the recommendation has been to maintain excellent hydration as a strategy to prevent the progression to oligo-anuric acute kidney injury and HUS. This strategy may need to be re-evaluated in a patient with SARS-CoV-2 infection or co-infection.

## Introduction

Seven coronaviruses cause human infections, mainly in children, who generally experience a mild transient upper respiratory tract infection (1). The spectrum of disease ranges from asymptomatic to severe and potentially lethal, mostly in seniors or those with co-morbidities. With the new millennia, three novel coronaviruses of zoonotic origin emerged: SARS-CoV-1, MERS-CoV, and SARS-CoV-2. Since its appearance in late 2019, the pathophysiology of COVID-19 infection is becoming clearer. In terms of the renal effects, a growing body of literature has chronicled the spectrum of disease related to SARS-CoV-2 infection. Initially the Acute Disease Quality Initiative work group highlighted a wide range of acute kidney injury (AKI) incidence. Nadim et. al later narrowed it to 20% in hospitalized patients, and to 50% in intensive care unit patients (2). Further, 50%–70% of those with AKI, particularly those with hematuria and proteinuria, needed renal replacement therapy (3). AKI significantly increased mortality risk (4).

As the pandemic continued, reports focused on the thrombotic microangiopathy (TMA) syndromes caused by SARS-CoV-2, such as hemolytic uremic syndrome (HUS). HUS, defined by the triad of acute kidney injury, thrombocytopenia, and anemia, is classically associated with Shiga toxin-producing *Escherichia coli* (STEC) (5). Treatment is defined by supportive care, as well as avoiding antibiotics (6). The role played by complement in the development of atypical HUS and COVID-19-associated TMA is underscored by the consistent finding of elevated C5b-9 levels. Merrill et. al, as well as Gavriilaki and Brodsky, hinted at the potential therapeutic role of C3 and C5 inhibitors (7, 8). Merrill also remarked on the similarities between the immune response in the SARS-CoV-2 cytokine storm and in other TMA syndromes (7). In addition to these theoretical pieces, several reviews and case reports have since established the clinical phenotype of TMA in hospitalized COVID-19 patients, where TMA or even a HUS picture is triggered by SARS-CoV-2 infection (9–13).

Regarding children infected with SARS-CoV-2, the literature has evolved over the past two years, depicting a growing number of COVID-19 infection clinical phenotypes. The first reports emerging from China (14) and other countries emphasized the often asymptomatic or mild nature of the disease compared to adults (15, 16). The most striking exception to this trend was the recognition in the summer of 2020 of a Kawasaki-like disease best known in North America as multisystem inflammatory syndrome in children (MIS-C). This syndrome, which presents as a late-phase manifestation of the disease, is attributed to the simultaneous decrease in viral replication and a crescendo inflammatory response, akin to that seen during a cytokine storm (17, 18). Other small studies, such as that by Oualha et.al, described the pediatric spectrum of serious COVID-19 disease involving the kidneys and other organ systems (19). Recently, a couple of publications report on the occurrence of HUS caused by COVID-19, similar to that previously described in adults (12), and on several children with known complement mutations who had been quiescent until SARS-CoV-2 infection triggered relapses (13).

In the United States, Oregon has an annual incidence above the national average for culture confirmed STEC infections (20), and cases have been steadily increasing over the past ten years, with 354 reported infections in 2019 (21). This includes O157, the most common serotype identified in diarrhea-associated HUS worldwide, but also a growing number of non-O157 serotypes. For this reason, Oregon is uniquely positioned to discuss the impact of COVID-19 and HUS.

## Case descriptions

There are two children's hospitals in Portland, which serve Oregon and southwest Washington, or roughly 875,000 children under age 18. These cases were drawn from these hospitals (see Table 1), IRB determined the case series to be exempt, and parental consent was obtained prior to submission.

**Table 1:** Case comparisons.

	Case #1	Case #2
Labs:		
COVID-19	PCR^a^ (+) Antibody not measured	PCR (+) Antibody (−)
Stool Culture	Negative STEC^b^	Positive STEC
Shiga toxins	Negative for both toxins 1 & 2	Positive for both toxins 1 & 2
Hemoglobin	3.1 g/dl	11.2 g/dl
Platelets	13,000 mm^3^^c^	31,000 mm^3^
LDH^c^ (peak)	4,861 U/l	3,101 U/l
Creatinine	8.50 mg/dl	5.01 mg/dl
C3	Normal	Normal
C4	Normal	Normal
CH50	Normal	Not measured
C5b-9	Elevated	Elevated
Discharge Creatinine	0.8 mg/dl	11.04 mg/dl
Outcome	CKD^d^ II with mild proteinuria	ESRD^e^

^a^ Polymerase chain reaction.

^b^ Shiga toxin-producing *Escherichia coli.*

^c^ Lactate dehydrogenase.

^d^ Chronic kidney disease.

^e^ End-stage renal disease.

Case #1 is a previously healthy ten-year-old, who had had an appendectomy six days prior to presentation (her final pathology showed a normal appendix). She had been discharged home with ongoing vomiting, lethargy, and bloody diarrhea. At the time of surgery, she had tested negative for COVID-19 by PCR, and her preoperative laboratory tests were all normal including a hemoglobin of 11.6 g/dl, platelets 214,000 mm^3^, as well as creatinine 0.40 mg/dl. After discharge she had ongoing intermittent “black and tarry” loose stools which prompted her second emergency room visit. At that time, she was oligo-anuric and her laboratory tests demonstrated severe anemia (hemoglobin 3.1 g/dl), thrombocytopenia (13,000 mm^3^), and smear with schistocytes (5–10 cells/HPF). Her creatinine was 8.5 mg/dl and her BUN was 143 mg/dl; her peak LDH was 4,861 U/l and she tested positive for COVID-19 PCR, but she was STEC negative. Her C3, C4 and CH50 were normal, and her C5b-9 was elevated. Her atypical HUS genetic panel found a heterozygous missense common variant in the plasminogen gene. She required ten days of hemodialysis, several blood transfusions, and antihypertensive therapy. She was discharged home on amlodipine with an eGFR that places her in CKD stage II. Despite several attempts, she has not returned for follow up care.

Case #2 is a previously healthy six-year-old who presented after four days of bloody diarrhea. He was COVID-19 PCR positive on admission and tested negative for COVID-19 IgG antibodies. On admission his inflammatory markers were elevated (C-reactive protein 3.61 and erythrocyte sedimentation rate 21), but all other labs were reassuring, including a negative stool culture and negative Shiga toxins. A contrast computed tomography scan showed pancolitis, and an ultrasound demonstrated large echogenic kidneys. The next day, he developed anemia (hemoglobin 11.2 g/dl). Three days later he developed a fever, thrombocytopenia (31,000 mm^3^), and anuria. No schistocytes were seen on his smear. His creatinine was 5.01 mg/dl, up from 0.4 mg/dl on admission. His LDH was 3,100 U/l, and his D-dimer was 18,130 ng/ml. Repeat Shiga toxin testing was positive for both toxins one and two. C3 and C4 were normal, and C5b-9 was elevated. His ADAMTS13 activity was 53%, and an atypical HUS genetic panel was negative for genetic variants. He started hemodialysis and received Tocilizumab for treatment of his COVID-19 infection, which coincided with resolution of his thrombocytopenia. He developed hypertension and required two antihypertensive agents (amlodipine and clonidine), which he continued on discharge. He was anticoagulated until discharge. After three weeks, despite an adequate urine output, his AKI remained. Four weeks after admission he was discharged to the outpatient hemodialysis unit. He has been dialysis dependent for approximately 10 months, currently remains dialysis dependent with end-stage renal disease (ESRD), and has been referred for transplant evaluation.

## Discussion

We aim to highlight a few points for the general pediatrician who is at the forefront when evaluating patients with diarrhea and possible HUS. First, SARS-CoV-2 is potentially a new infectious etiology to include in the differential diagnosis of diarrhea-associated HUS, either as the sole agent or concurrent with a STEC infection. If concurrent, the patient is fighting two pro-thrombotic conditions. This underscores the importance of testing for both infections with a fecal PCR panel and a COVID-19 PCR or antibody screen (as appropriate). Second, maintaining excellent hydration is the longstanding recommendation to prevent STEC gastroenteritis from progressing to oligo-anuric AKI. This strategy may need to be re-evaluated in COVID-19 PCR positive patients. In this context, the patient may be in different stages of COVID-19 disease that require different volume management strategies. A patient presenting early in the course may be in the volume-depleted early stage of the acute-mild disease; in this stage, the general advice of supporting adequate to generous hydration applies. If they present in the more severe acute stage when acute respiratory distress syndrome is more common, moderation with hydration is necessary. Worse still, the patient may be in the most critical stage of severe MIS-C, which mimics the leaky capillary state of a cytokine storm. In this scenario, judicious fluid management is required and early input from infectious disease, rheumatology, and nephrology is essential, usually best achieved by referring the patient to a tertiary center.

Geographical and historical awareness are crucial to understanding these cases. As one of the epicenters of STEC-associated HUS in the nation, our experience may differ from others. SARS-CoV-2 is the latest great mime, included in the differential diagnosis of almost every patient, reflecting our incipient yet growing knowledge of the pathophysiology of this infection. We are, for example, still learning about the association between angiotensin-converting enzyme 2 (ACE2) receptor expression in different organs. It is interesting to note that ACE2 receptor expression in the nasal epithelium is age dependent, and its lower expression in the young may be one of the reasons for the lower incidence of COVID-19 disease in children (22). It is possible the associations drawn here are simply coincidental; yet these cases are consistent with the current understanding of the pathophysiology of COVID-19 disease.

As with most case reports, the analysis is retrospective and the strict criteria that apply to prospective trials in terms of sample collection and clinically relevant information is not always achieved. We were, however, impressed by this cluster of unusual HUS cases during the second year of the pandemic, which also coincided with a relaxation in the social distancing rules and a sharp increase in pediatric COVID-19 cases. In our view, this warranted sharing our observations to facilitate increased awareness in the pediatric community.

## Data Availability

The original contributions presented in the study are included in the article/Supplementary Material, further inquiries can be directed to the corresponding author/s.
